# International validation of meaningfulness of postural sway and gait to assess myeloneuropathy in adults with adrenoleukodystrophy

**DOI:** 10.1002/jimd.12753

**Published:** 2024-05-25

**Authors:** Hemmo A. F. Yska, Bela R. Turk, Ali Fatemi, Jordan Goodman, Marije Voermans, Dan Amos, Man Amanat, Stephanie van de Stadt, Marc Engelen, Amena Smith‐Fine, Jennifer Keller

**Affiliations:** ^1^ Emma Children's Hospital, Department of Neurology and Pediatric Neurology, Amsterdam Leukodystrophy Center, Amsterdam University Medical Centers University of Amsterdam Amsterdam The Netherlands; ^2^ Kennedy Krieger Institute Baltimore Maryland USA; ^3^ Department of Neurology Johns Hopkins School of Medicine Baltimore Maryland USA; ^4^ Department of Physical Medicine & Rehabilitation Johns Hopkins School of Medicine Baltimore Maryland USA

**Keywords:** adrenoleukodystrophy, adrenomyeloneuropathy, clinical trial, gait, sway, validation, wearable devices

## Abstract

**Background:**

The most common manifestation of X‐linked adrenoleukodystrophy (ALD) is a slowly progressive myeloneuropathy, which leads to imbalance and gait disturbances. The variable progression of the disease complicates evaluation of its progression rate. Wearable sensors allow for easy and frequent balance and gait collection. This study reports baseline data from a longitudinal study on the quantitative assessment of balance and gait with wearable sensors and their clinical relevance.

**Methods:**

Data were collected from adult patients in two institutions. Postural body sway and gait parameters were measured using accelerometers. Disease severity was measured by the Expanded Disability Severity Scale (EDSS). Falling frequency and quality of life (QOL) were collected in men. The relationship between sway and gait variables and EDSS score, participants' use of a walking aid, and falling frequency was evaluated.

**Results:**

One hundred twenty individuals with ALD were included. Sway variables significantly differentiate participants' assistive device use. Sway and gait variables were correlated to the EDSS in both sexes. Both gait speed and sway were correlated with falling frequency in men from one institution. Select QOL subscores were correlated with the EDSS in males from one institution. Accelerometry generated comparable results across sites.

**Discussion:**

This study confirms the clinical correlation between spinal cord disease and imbalance and gait in ALD. For the first time, this study shows clinically meaningful relationships for sway and gait with use of an assistive device, falling frequency and QOL. Wearable accelerometers are a valid means to measure sway and gait in ALD. These measures are promising outcomes for clinical trial designs to assess myeloneuropathy in ALD and to monitor disease progression in individuals.

## 1 INTRODUCTION

Adrenomyeloneuropathy (AMN) is a neurodegenerative disorder with no market approved therapy, and the most common variant of X‐linked adrenoleukodystrophy (ALD). AMN disease progression is slow, highly variable and biomarkers remain elusive. The diagnostic prevalence of ALD is rising due to the adoption of ALD newborn screening on most state screening panels within the last decade. All ALD males will eventually develop the AMN phenotype.^1^ Most ALD females, earlier assumed to be asymptomatic carriers, also develop AMN symptoms.^2^ Pathologically, AMN is a distal axonopathy that affects the dorsal column and corticospinal tracts, characterized primarily by lower extremity weakness and abnormalities in vibration sensation, spasticity, balance, and walking.^3^, ^4^ The symptoms severely impair patients' quality of life, especially daily physical functioning.^5^, ^6^

There is an unmet need for clinicians to quantify disease severity, monitor progression, and thereby provide prognostic guidance or precision therapeutic management that addresses these symptoms.^7^ Variability in progression rate and symptom pattern also poses a critical challenge for clinical trial design, specifically in patient selection given the limited population and trial duration required to detect significant change in the commonly used clinical rating scales.^7^

Postural body sway and gait assessments provide reproducible and continuous results and have therefore been proposed as an alternative to ordinal disability scales for measuring AMN disease severity and progression.^8^, ^9^, ^10^ Impaired walking is associated with weakness and overall disability as measured by the EDSS.^11^ Balance, defined by sway amplitude during static standing on a force plate, is impaired in both males and females with AMN, and is related to both sensory loss and decreased hip strength.^11^, ^12^ Attempts to quantify AMN progression have demonstrated that force plate sway amplitude worsens for both males and females over 2 years, whereas walk speed declines for males only, reflecting either a difference in sensitivity of these measures, or a physiological difference in progression patterns of the sexes.^4^, ^13^ What is not known about these measures is their direct relevance to the quality of life of people with ALD.

Sway and gait testing in neurological disorders has traditionally been performed in a motion lab with specialized equipment, requiring a high degree of operator training, expense and technical knowledge to process and analyze. Wearable sensors may provide an alternative to these techniques, as they are not bound by these limitations and they can be deployed for supervised remote assessments in regions where patients have limited access to specialized centers. Removing barriers to participation due to burdens of travel, time, cost, may also improve longitudinal study attrition. Validity of wearable accelerometers has been established in comparison with gold standard equipment in the motion lab such as the force plate and 3D infrared motion capture systems. Furthermore, they have been used to characterize impairment and progression in a variety of neurological disorders.^8^, ^14^, ^15^, ^16^, ^17^

The goals of this study were (1) to examine how sway and gait, measured with wearable sensors, reflect cross‐sectional disease severity in males and females with AMN, and (2) investigate the clinical meaningfulness of sway and gait in AMN, which represents an unmet need to advance preparedness for clinical trials.

## 2 METHODS

### 2.1 Participants and procedures

Adult patients with a diagnosis of ALD were included at two sites: Amsterdam University Medical Centers (AMC) in the Netherlands, and the Kennedy Krieger Institute (KKI), Baltimore, MD. Sway and/or gait was assessed at least once between 2020 and 2022. All variables were sampled at one time point. Patient exclusion criteria were inability to stand independently, a diagnosis of active cerebral ALD or any comorbidity with a known direct effect on sway. All patients provided informed consent (medical ethical decisions AMC: 2018_310; KKI: IRB00150619).

### 2.2 Data collection

Sway and gait were assessed with the Opal system (APDM, Portland, OR). Accelerometers were placed around the waist and dorsum of the feet of each participant. Mobility Lab software (APDM) was used for data post‐processing and analysis. Postural body sway was collected over the course of 30 s, in four standing paradigms: feet apart—eyes open (FAEO), feet apart—eyes closed (FAEC), feet together—eyes open (FTEO) and feet together—eyes closed (FTEC). Gait was assessed by a 6‐min walk test at fast‐walking pace (AMC) or by a 2‐min walk test at self‐selected walking pace (KKI). Walking tests at the Amsterdam site were conducted during a routine outpatient clinic visit. Walking tests from patients at KKI were either performed during a clinic visit, or data were collected at home while under direct supervision of research staff.

### 2.3 Outcomes

#### 2.3.1 Sway and gait

The OPAL system accelerometers and Mobility Lab analyses sample and derive 33 sway‐ and 35 gait variables.^18^ All variables were included in the analyses in this study, but emphasis was placed on a selection of outcome measures that have been associated with spinal cord pathology in ALD.^4^, ^11^ A priori batch analysis was performed on all sway and gait variables. The presented statistical results were chosen based on preformulated hypotheses. The following variables were selected:

##### Sway

*RMS sway* is the root mean square (RMS) of the sway angle in both the coronal and sagittal planes (Supplementary material 1). *Sway area* is the area of an ellipse covering 95% of the sway angle in the coronal and sagittal planes. *Path length* is the total length of the sway path in the transverse plane.

##### Gait

*Toe‐off angle* is the angle of the foot sole versus the mechanical axis of the lower leg as it leaves the floor at push off, the angle of the foot when flat is zero (Supplementary material 1). *Gait speed* is the forward movement speed, calculated using the distance traveled during the gait cycle divided by the gait cycle duration. The full list of variables and definitions generated by the OPAL system can be found elsewhere.^18^

#### 2.3.2 Disease severity—Global scores

Spinal cord disease‐related disability was assessed using the Expanded Disability Status Scale (EDSS). The EDSS is the current gold standard global scoring system for the myeloneuropathy phenotype. A higher score indicates more severe disease.

#### 2.3.3 Use of an assistive walking device

An EDSS ≥6.0 indicates that patients require unilateral assistance to walk at least 100 m. Participants' use of an assistive device for walking included walking canes and other mobility assistive devices.

#### 2.3.4 Falling frequency

Male patients in the Dutch ALD cohort were sent a questionnaire every 2 weeks (automated questionnaire from the Castor EDC) during a 6–9 month period asking whether they had fallen within this period of time. In order to account for response rate, we calculated the proportion of surveys where patients indicated they fell as a percentage of the total number of surveys they returned. For example, if a patient only returned the questionnaire six times, but indicated having fallen every time, their falling frequency would be calculated as 100%. If sampled 12 times, and a patient reported falling at six timepoints, their falling frequency was calculated as 50%.

#### 2.3.5 Quality of life

QOL was assessed using the Short Form health survey (SF‐36).

### 2.4 Statistical analysis

Statistical analyses were performed using R‐studio, Python 3.10.13, packages including Stats 1.11.12, matplotlib 3.7.2, Scikitlearn 3.12.0, Belastats 0.11 and Belacrunchtime 0.15. Sway and gait comparison were performed for use versus non‐use of an assistive device. Independent samples *t*‐tests, and analysis of variance (ANOVA) were used to test differences between means. Relationships between sway, gait, EDSS and SF‐36 outcomes were performed using least squares linear regression and generalized mixed linear models. A *p* < 0.05 was considered statistically significant for all tests. Uncorrected p‐values reported from hypothesis driven analyses are reported in this paper.

#### 2.4.1 Outlier analysis

Outliers were removed if computer‐generated or trial site supervisor documentation from the time point indicated the participant was unable to complete a trial or specific paradigm. Statistical outliers without documentation of inability to complete a trial were not excluded.

## 3 RESULTS

### 3.1 Demographics

**FIGURE 1:**
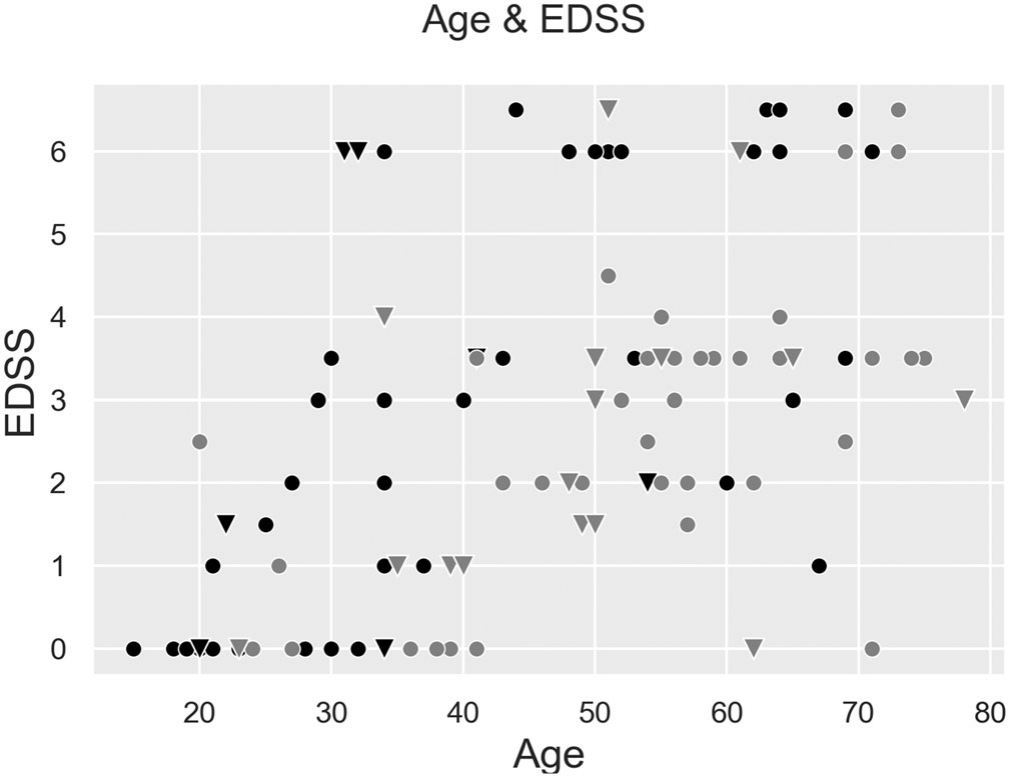
Relationship between age and Expanded Disability Status Scale (EDSS). ALD patients male (black) and female (gray), EDSS and age at AMC (dots) and KKI (triangles).

One hundred twenty patients who had a confirmed genetic diagnosis of ALD were prospectively recruited at two sites, Amsterdam University Medical Centers and KKI. Ninety‐two patients at the Amsterdam site and 28 patients at the KKI site were included. Fifty‐nine males (49%) and 61 females (51%) were included. The mean age of all patients was 48 ± 20 years with a mean EDSS of 2.9 ± 0.2 points (Figure 1). The mean male age was 42 ± 18 years and 55 ± 15 years for females. The mean EDSS was 2.9 ± 2.6 for males and 2.8 ± 1.8 for females.

### 3.2 Gait

Forty‐one males and 42 females with ALD performed walking tests. At AMC, 32 Males and 25 females performed walking tests. At KKI: 9 males and 17 females were able to complete the walking tests.

### 3.3 Sway

A total of 52 males and 58 females completed at least one postural body sway trial. Four paradigms, Feet apart—eyes open (FAEO), Feet apart—eyes closed (FAEC), Feet together—eyes open (FTEO) and feet together—eyes closed were performed. At AMC: FAEO (43 males, 39 females), FAEC (37 males, 33 females), FTEO (43 males, 34 females), FTEC (35 males, 33 females). At KKI: FAEO (9 males, 19 females), FAEC (9 males, 18 females), FTEO (9 males, 18 females), FTEC (8 males, 17 females). Twenty‐two of 241 total sway trials were excluded from the dataset due to documented inability to complete the trial.

### 3.4 Sway and gait distinguish between users and non‐users of an assistive device

**FIGURE 2:**
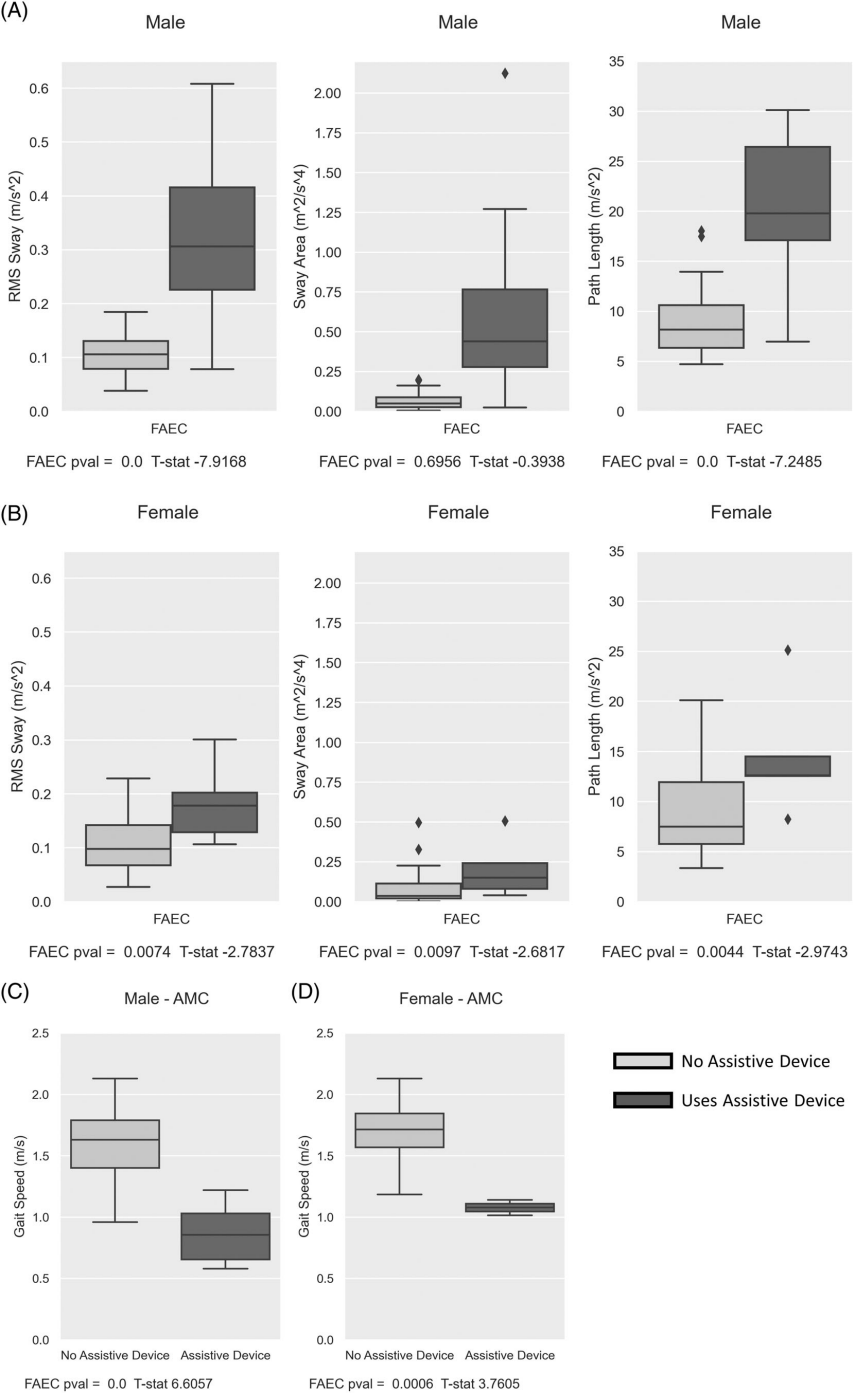
Use versus non‐use of assistive device. Use (light) versus non‐use (dark) of assistive device for sway area, root mean square sway (RMS sway) and path length for the feet apart—eyes closed (FAEC) paradigm for male (A) and female (B) patients from the AMC and KKI combined. Use (light) versus non‐use (dark) of assistive device for gait speed for male (C) and female (D) patients from the AMC.

When comparing between use versus non‐use of an assistive walking device, sway in the FAEC paradigm was significantly different in males and females for RMS sway, sway area and path length (males all *p* < 0.001; females *p* = 0.007; *p* = 0.001; *p* = 0.004, respectively) (Figure 2A,B). Both “eyes closed” paradigms resulted in the highest mean sway in both sexes. Female patients had lower mean sway than males. Gait speed also showed significant differences in both sexes (*p* < 0.001) (Figure 2C,D).

A threshold for FAEC RMS sway at 0.2 m/s^2^ for men with ALD displays a Sensitivity of 1.0 and Specificity of 0.97 for stratifying use versus non‐use of assistive device. One patient which uses a device falls under 0.2 m/s^2^. Of note is that this patient's other quantitative sway measurements fall within the range of non‐device users, and use of device is by patient choice. No comparable threshold was seen for female patients with ALD for sway, however gait speed showed no overlap between device versus non‐device use.

### 3.5 Sway and gait correlate with disease severity

**FIGURE 3:**
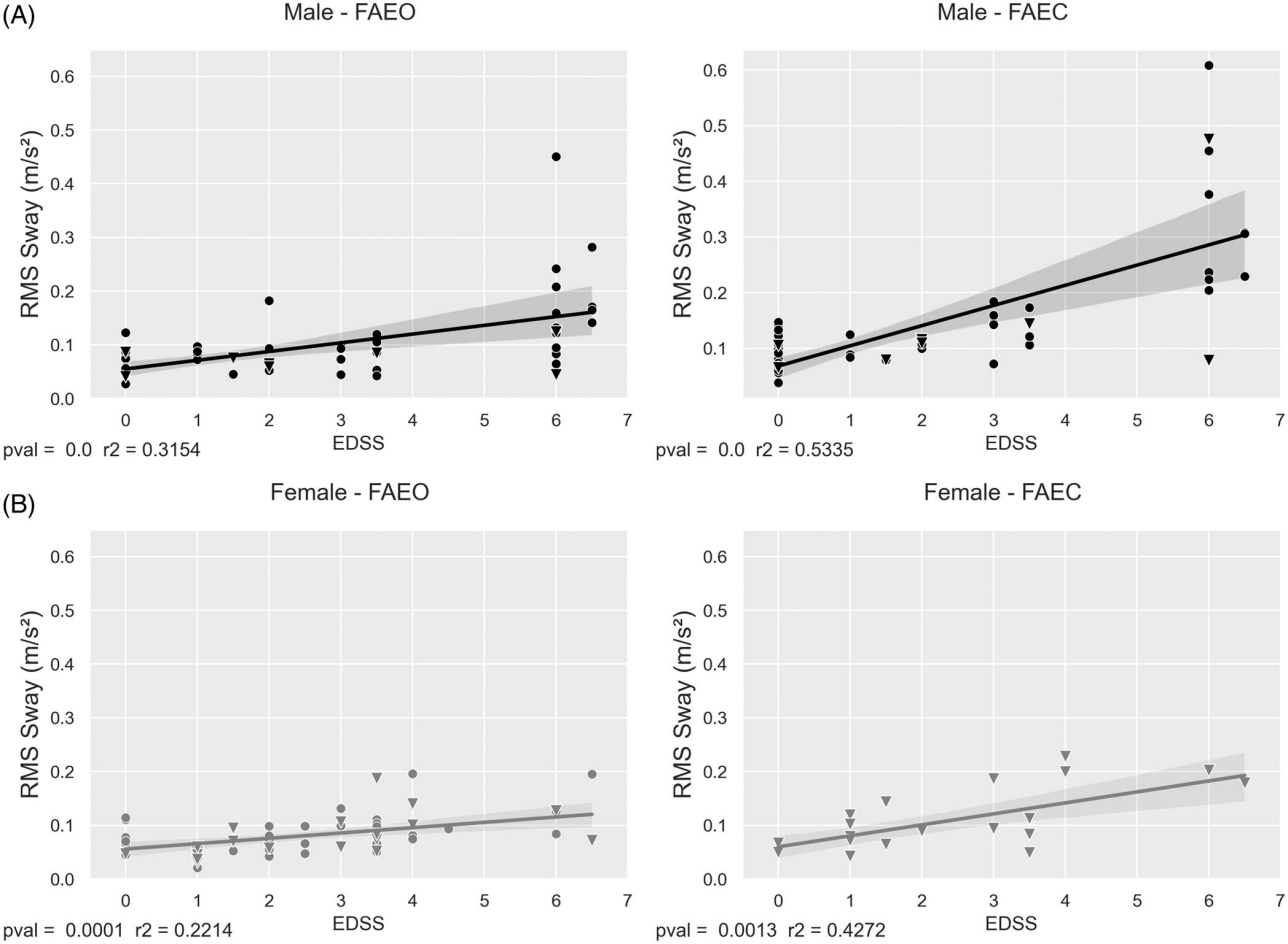
Linear regression between sway and Expanded Disability Status Scale (EDSS). Linear regression for RMS sway versus EDSS for males (A) and females (B), at AMC (dot) and KKI (triangle). FAEO, feet apart—eyes open; FAEC, feet apart—eyes closed.

In males, of the sway variables under analysis, the strongest correlations were seen for EDSS and RMS sway (Figure 3A). In females, significant correlations with lower coefficients than males were seen. Statistically significant correlations were seen in both FAEO and FAEC for males (FAEO: *R* ^2^ = 0.315, *p* < 0.001; FAEC: *R* ^2^ = 0.534, *p* < 0.001) and females (FAEO: *R* ^2^ = 0.221, *p* < 0.001; FAEC: *R* ^2^ = 0.427, *p* = 0.001). Higher sway was related to higher disability, and a high EDSS showed a higher range of sway in men.

**FIGURE 4:**
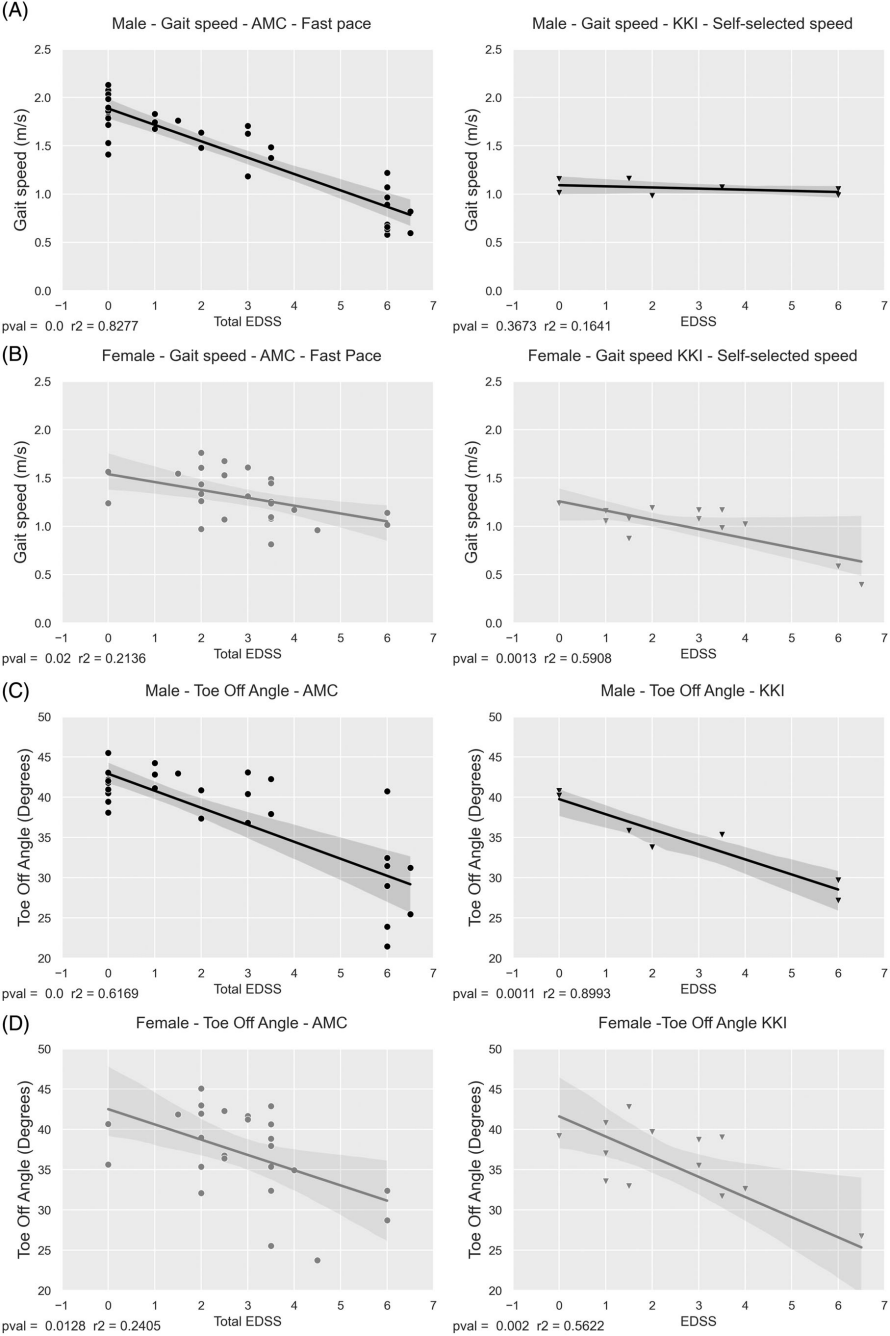
Linear regression between gait and Expanded Disability Status Scale (EDSS). Linear regression of EDSS versus gait speed in (A) males and (B) females and toe‐off angle in (C) males and (D) females. Patients from KKI (triangles) and AMC (dots). For AMC patients, gait was obtained during a 6‐min walk test (fast walking pace). For KKI patients, gait was obtained during a 2‐min walk test (self‐selected walking pace).

Gait speed showed strong correlations with EDSS in male patients from the AMC who performed a 6‐min walk test (fast‐walking pace) (*R* ^2^ = 0.828, *p* < 0.001), but not in male patients from KKI who performed a 2‐min walk test (self‐selected walking pace) (*R* ^2^ = 0.164, *p* = 0.367) (Figure 4A). Significant correlations were found in female patients from both institutions for both walking tests (AMC: *R* ^2^ = 0.214, *p* = 0.02; KKI *R* ^2^ = 0.591, *p* = 0.001) (Figure 4B). Gait speed decreased as EDSS increased. Toe‐off angle correlated significantly with EDSS in patients from both AMC and KKI. Toe‐off angle decreased as EDSS increased. Regression coefficients were higher for males (AMC: *p* < 0.001, *R* ^2^ = 0.617; KKI: *p* = 0.001, *R* ^2^ = 0.899) (Figure 4C) than for females (AMC: *p* = 0.013, *R* ^2^ = 0.241; KKI: *p* = 0.002, *R* ^2^ = 0.562) (Figure 4D).

### 3.6 Sway and gait correlate with falling frequency in males

**FIGURE 5:**
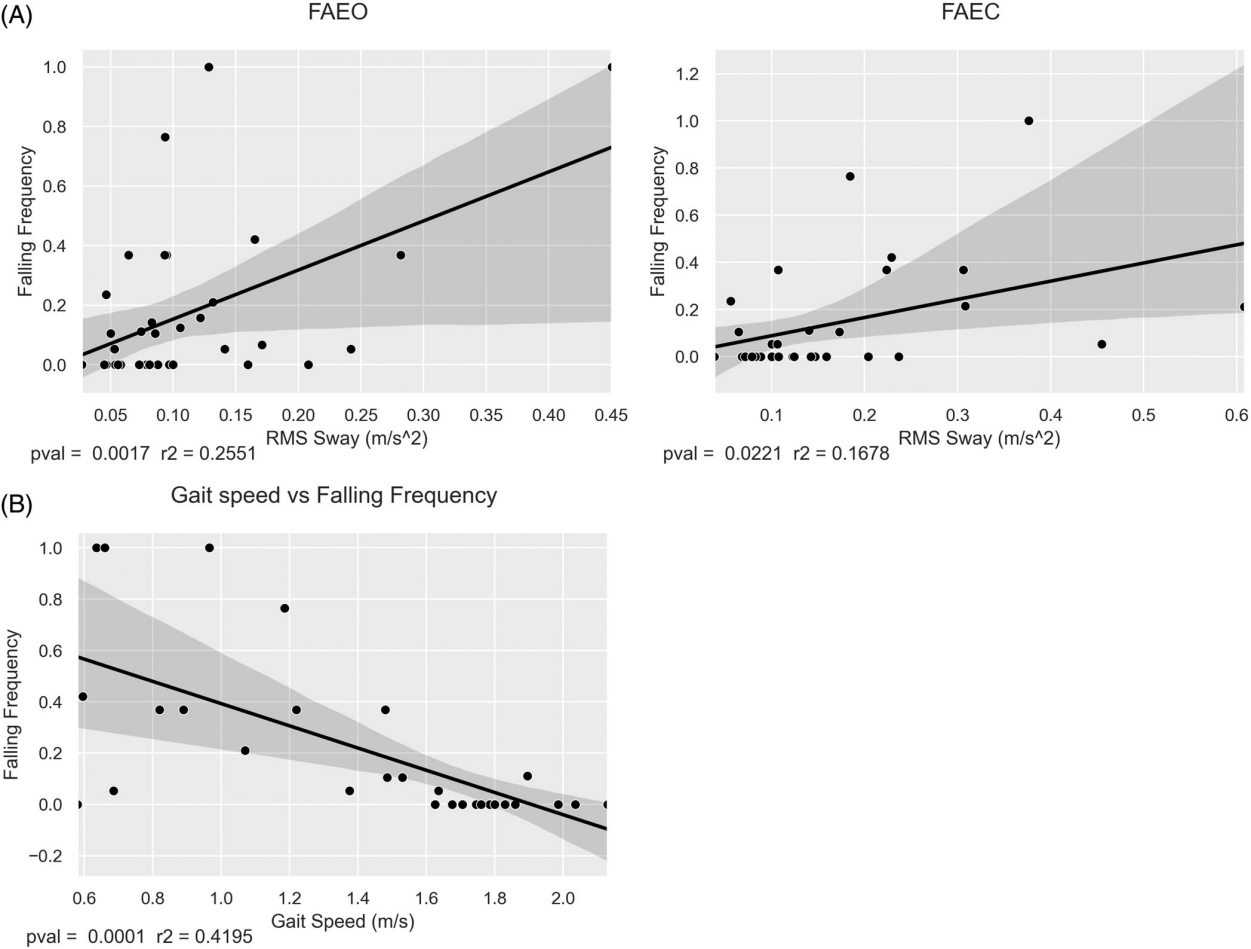
Linear regression between sway and gait and falling frequency. Linear regression of RMS sway and gait speed versus falling frequency in males from the Amsterdam UMC cohort. Falling frequency increases as RMS sway increases and as gait speed decreases.

Falling data (*“Did you fall within the last two weeks?”*) were collected bi‐weekly over a period of 9 months (19 time points) at the AMC site from 39 male patients. Mean response rate was 15.4 time points (81%), std 5.5 (21%). Falling Frequency was calculated as a percentage of each individual's total responses (fall vs. non‐fall). RMS Sway and gait speed correlated with falling frequency. RMS sway showed a significant correlation in the paradigms FAEO (*R* ^2^ = 0.255, *p* = 0.002) and FAEC (*R* ^2^ = 0.168, *p* = 0.022), and between gait speed and falling (*R* ^2^ = 0.420, *p* < 0.001) (Figure 5).

### 3.7 Sway and gait correlate with SF‐36 quality of life subscores in males

**FIGURE 6:**
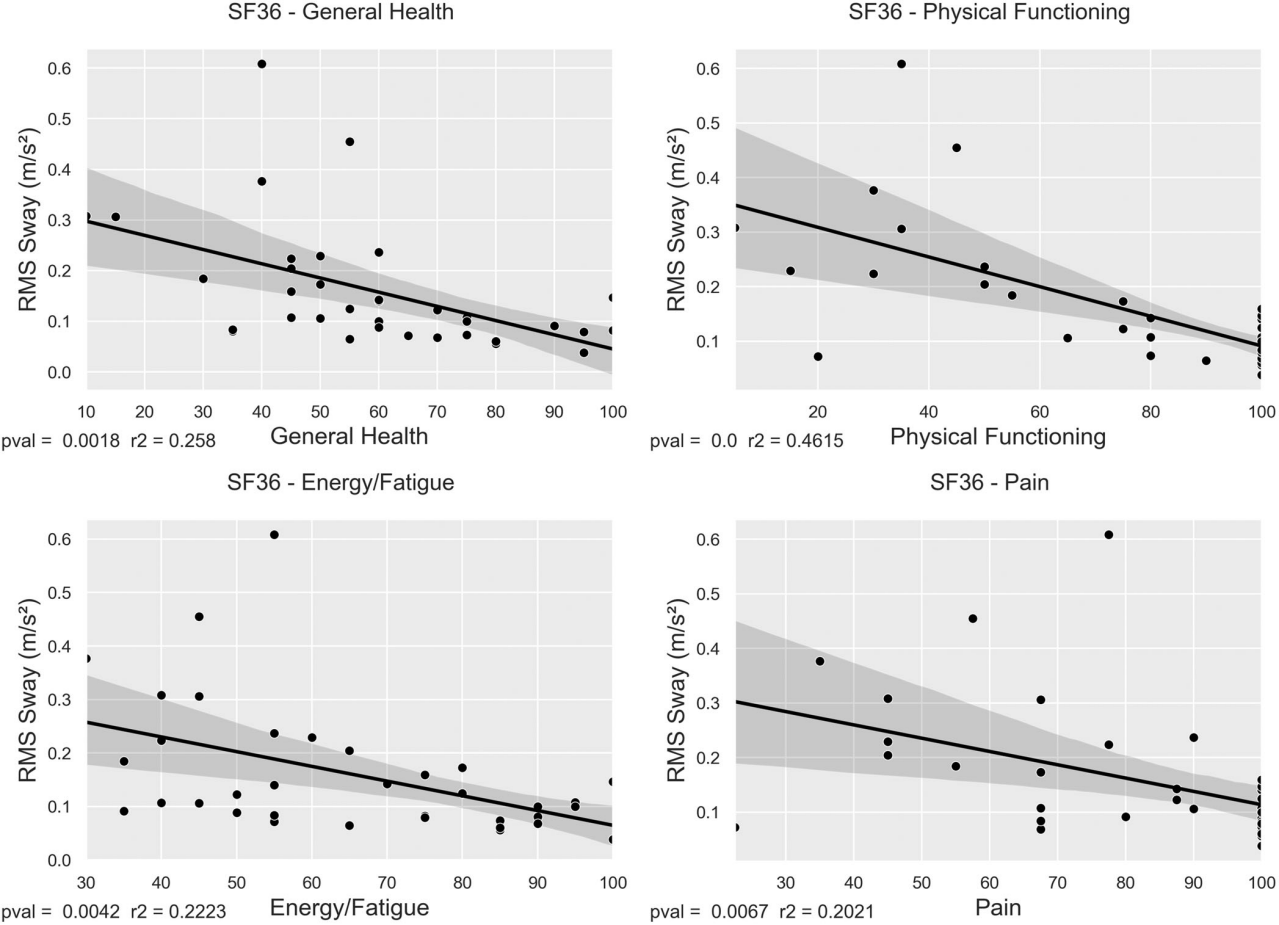
Linear regression between sway and quality of life. Linea regression between RMS sway and 36‐item Short Form Health Questionnaire (SF‐36) subscores (general health, physical functioning, energy/fatigue and pain) in males from the Amsterdam UMC cohort.

SF‐36 data were collected from 35 male ALD patients at the AMC site, who also completed postural sway testing. Linear regression was performed between sway and SF‐36 subscales. Significant correlations between RMS sway in the FAEC paradigm and several SF‐36 subscales were seen in the domains: Physical functioning (*R* ^2^ = 0.462, *p* < 0.001), general health (*R* ^2^ = 0.258, *p* = 0.002), energy/fatigue (*R* ^2^ = 0.222, *p* = 0.004) and pain (0.202, *p* = 0.007). Correlations for the other 5 subscales were not significant. Lower SF‐36 scores were associated with higher sway (Figure 6).

## 4 DISCUSSION

In this study, we show clinical meaningfulness of sway and gait. Two pivotal symptoms of people with ALD, imbalance and gait dysfunction, were measured with accelerometry. The results of these measurements were related to disease severity as measured by the EDSS in both males and females. Our results indicate differences in the balance and gait impairment between sexes. The accelerometers generated postural sway results within the same range at both institutions. Shown for the first time, postural sway differentiated between individuals who do and do not require an assistive device for ambulation. Falling frequency and quality of life, which were queried in men at the AMC, related to specific parameters of postural body sway and gait dysfunction. These results represent the baseline analysis of a multi‐center longitudinal study investigating sensorimotor impairment in adults with AMN.

The myelopathy of ALD is characterized by a dying‐back axonopathy of the corticospinal tracts and dorsal columns.^3^ The resulting spasticity, weakness, and proprioceptive dysfunction result in worsened balance and gait unsteadiness. A recent study suggested that cortical systems including the basal forebrain may be involved in addition to the spinal cord lesions. Cholinergic neurons of the basal forebrain stained more positive for the ABCD1 protein than neurons of the motor cortex thus indicating a pathological correlate.^19^ Still, it is assumed that spinal cord dysfunction is a core element of the pathology in ALD. Disease severity in ALD has often been measured by the EDSS, which quantifies sensory and motor dysfunction on an ordinal scale. The data in this study show that those with a higher EDSS score have higher sway and demonstrate slower walk speed, consistent with prior reports.^11^ Toe‐off angle also correlated well to the EDSS. Multiple pathological mechanisms may explain this result. A change in toe‐off angle could be the effect of a decreased walking speed or impairments like spasticity or ankle weakness which would result in less range of motion during the gait cycle. All of these are impairments are observed in ALD. Sway testing paradigms included “eyes open—feet apart” (FAEO) versus “eyes closed—feet apart” (FAEC). By removing visual input in the eyes closed paradigms, postural balance becomes reliant on proprioceptive functions of the dorsal column. In ALD, where proprioception is inadequate to maintain posture, the eyes closed paradigms resulted in higher sway than the eyes open paradigms. A further condition, “feet together” (FTEO) allowed for an even more challenging test by narrowing the base of support. The most challenging paradigm: “eyes closed—feet together” (FTEC) was too challenging for some people with severe disease to complete. Using four testing paradigms can for this reason allow for the monitoring of patients with a diverse range of disease. The most challenging paradigm FTEC may be sensitive to small changes in early‐symptomatic individuals. Sway in the FAEC paradigm may be more appropriate for patients with severe disease as some may not be able to perform tests in the FTEC paradigm. Furthermore, differences between males and females may dictate the value of specific sway and gait paradigms or variables, as males in this study demonstrate higher sway values than females when closing their eyes at a given disease severity score. Future studies should further investigate the practical use of sway, its testing paradigms and the choice of variables for subpopulations.

The 6‐min walk test is one of the most commonly used and validated techniques to evaluate disability in ALD and other neurological diseases.^20^, ^21^, ^22^, ^23^ Although walking distance is a clinically relevant outcome, it is influenced by factors such as motivation, comorbidity and the use of a walking aid.^24^, ^25^, ^26^ Additionally, there is a floor and ceiling effect. The walking tests of patients with mild disease are often normal as they are still able to compensate for their disability. Patients with severe disease, on the other hand, may not be able to perform these tests at all. In the current work, for both the fast‐paced 6‐min walk test and self‐selected pace 2‐min walk test, quantitative measures of gait were found to be sensitive for spinal cord disease severity. Gait speed and toe‐off angle correlated well to the EDSS. Men with lower gait speed had higher falling frequencies and gait speed differentiated between users and non‐users of a walking aid in both sexes. The 2‐min walk test requires only a 20‐ft walkway versus the 6‐min walk test 100‐ft walkway, making this test more accessible when space is limited (e.g., home, clinic) or when individuals experience exercise intolerance due to fatigue or pain.

Measuring postural sway by accelerometry offers an expanded means for functional assessment in ALD. Sway trials are less influenced by motivation and can be performed by a wider range of individuals with disability than walking tests. Sway measurements can also be collected remotely, which is particularly helpful in regions where patients have limited direct access to specialized healthcare providers. Findings from this study support sway as a clinically meaningful outcome measure. First, the variable RMS sway correlated well to the EDSS at both sites. Second, in both men and women with ALD, selected sway variables distinguished between users and non‐users of an assistive walking device at both sites. Third, sway correlated to falling frequency in males in the Dutch ALD cohort. Falls can have a great impact on daily functioning and have previously also been correlated to sway and gait.^27^ Finally, sway correlated to four subscores of the quality of life questionnaire SF‐36 in Dutch males: physical functioning, general health, energy and fatigue, and pain. Sway did not correlate to any of the mental health domains of the SF‐36. Previous studies reported similar findings with the highest disease impact of ALD on the physical functioning domain. A recent study did however also identify effects on mental health.^5^

Strengths of this study include the large sample size given a rare disease, standardized use of sway measurement equipment, and a comparison of populations from two countries. Some limitations also apply to this work. Although the EDSS is a highly utilized disease severity score in ALD, it was originally designed for multiple sclerosis and may therefore not fully represent the disease characteristics or underlying pathology in ALD. This underscores the need for clinically relevant outcome measures that are disease‐specific and sensitive to change. Use of an assistive device as an outcome measure is also challenging, as it is influenced by an individual's choice. For gait, the results of the 2 walk tests (2MWT and 6MWT) could not be combined. The discrepancy in correlation coefficients between the 2MWT and the 6MWT may be the result of different population sizes or differences in sensitivity of the testing paradigms for specific pathological aspects of ALD. Ongoing studies with gait measurement using both tests have been initiated at both sites. Although sway and gait were previously found to worsen with age, it was not corrected for in the analyses.^28^, ^29^ Van Ballegoij et al. concluded that the majority of sway variables in their study remained significantly correlated to EDSS after correction for age.^13^ Other background variables such as height and weight may also have influenced sway and gait.^28^, ^30^ Dutch participants are expected to be taller than American participants but there was insufficient data to draw this conclusion. Anthropomorphic measurements have been added to the standard data collection procedure at our institutions and corrections will be applied in future studies. It should be noted that the confounding effect of these variables will be of less importance in intra‐individual follow‐up. Although the Opal system was previously found to be a reliable system,^31^ this study did not analyze reproducibility. A previous publication described intra‐correlation coefficients in healthy controls ranging from 0.60 to 0.89.^10^, ^32^ Future studies should explore intra‐patient variability in patients with ALD. The same holds true for validity. It can be argued that it is of lesser importance if wearables are able to calculate exact gait values if they are reliable within populations or individual patients. Nevertheless, measuring these variables in the correct way remains important. One recent study found that in some patients accelerometers measure a slower gait speed than when measured manually with a stopwatch in faster patients.^33^ Although this effect would in our publication sooner result in an underrepresentation of the correlation than an overrepresentation of the coefficients, future studies should further address the validity of gait speed when measured by the Opal sensors.

In conclusion, this cross‐sectional study shows that sway is a clinically meaningful outcome measure for myelopathy in ALD. Sway and gait differentiate between users and non‐users of an assistive device, correlate to falling frequency, and QOL scores. Use of wearable accelerometers can provide a means to expand monitoring at home. Longitudinal changes of these measurements should be explored to determine the degree of change needed to reflect efficacy of therapeutics in clinical trials.

## AUTHOR CONTRIBUTIONS

**Hemmo A. F. Yska**: Drafting of the manuscript; acquisition of data; analysis and interpretation of data. **Bela R. Turk**: Drafting of the manuscript; study design; analysis of data. **Ali Fatemi**: Revision of the manuscript; study concept and design; interpretation of data. **Jordan Goodman**: Acquisition of data. **Marije Voermans**: Acquisition of data. **Dan Amos**: Acquisition of data. **Man Amanat**: Analysis and interpretation of data. **Stephanie van de Stadt**: Acquisition of data. **Marc Engelen**: Revision of the manuscript; study concept and design. **Amena Smith‐Fine**: Drafting and revision of the manuscript; acquisition of data; study concept and design; interpretation of data. **Jennifer Keller**: Revision of the manuscript; acquisition of data; study concept or design; interpretation of data.

## FUNDING INFORMATION

This research was partially funded by a grant from Netherlands Organization for Scientific Research (NWO) (VIDI 016.196.310), partially funded by a grant from the NINDS/NCATs (U54NS115052), and NICHD (P50HD103538).

## CONFLICT OF INTEREST STATEMENT

B. Turk served as institutional consultant to Minoryx, Poxel Therapeutics, received travel expenses for conference attendance (ALD Connect, ULF). A. Fatemi serves a principal investigator of clinical trials sponsored by for Minoryx, and Viking Therapeutics, and has served as Institutional consultant to Swanbio, Poxel Therapeutics, Autobahn Therapeutics, Ionis Therapeutics, Affinia Therapeutics and holds a patent for a product licensed to Ashvattha Therapeutics unrelated to the topics discussed. M. Engelen received research support from SwanBio Therapeutics, Minoryx, BlueBirdBio and Autobahn Tx. Principal investigator for ADVANCE (Minoryx), Cygnet (SwanBio) and PROPEL (SwanBio). A. Smith‐Fine has served as a consultant to Ionis Therapeutics, and serves as a co‐investigator of clinical trials sponsored by Minoryx and Viking Therapeutics. J. Keller served as institutional consultant to Minoryx, Poxel Therapeutics, received travel expenses for conference attendance (ALD Connect, ULF). H. A. F. Yska, J. Goodman, M. Voermans, D. Amos, M. Amanat, S. van de Stadt declare no conflict of interest.

## ETHICS STATEMENT

All procedures followed were in accordance with the ethical standards of the responsible committee on human experimentation (institutional and national) and with the Helsinki Declaration of 1975, as revised in 2000 (5). Informed consent was obtained from all patients for being included in the study. Ethical approval for this study was given by the local medical ethical review committees of the Amsterdam UMC and the Kennedy Krieger Institute.

## Supporting information


**Data S1.** Supporting information.

## Data Availability

The data that support the findings of this study are available from the corresponding author upon reasonable request.
