# Supplementary material for: International validation of meaningfulness of postural sway and gait to assess myeloneuropathy in adults with adrenoleukodystrophy
Source: J Inherit Metab Dis. 2024 May 25;47(6):1336–47. doi: 10.1002/jimd.12753 (PMC11586604; doi:10.1002/jimd.12753)
Supplement: Supplementary file 1 — Data S1. Supporting information. [file JIMD-47-1336-s001.docx]

**Visual representation of postural body sway testing**


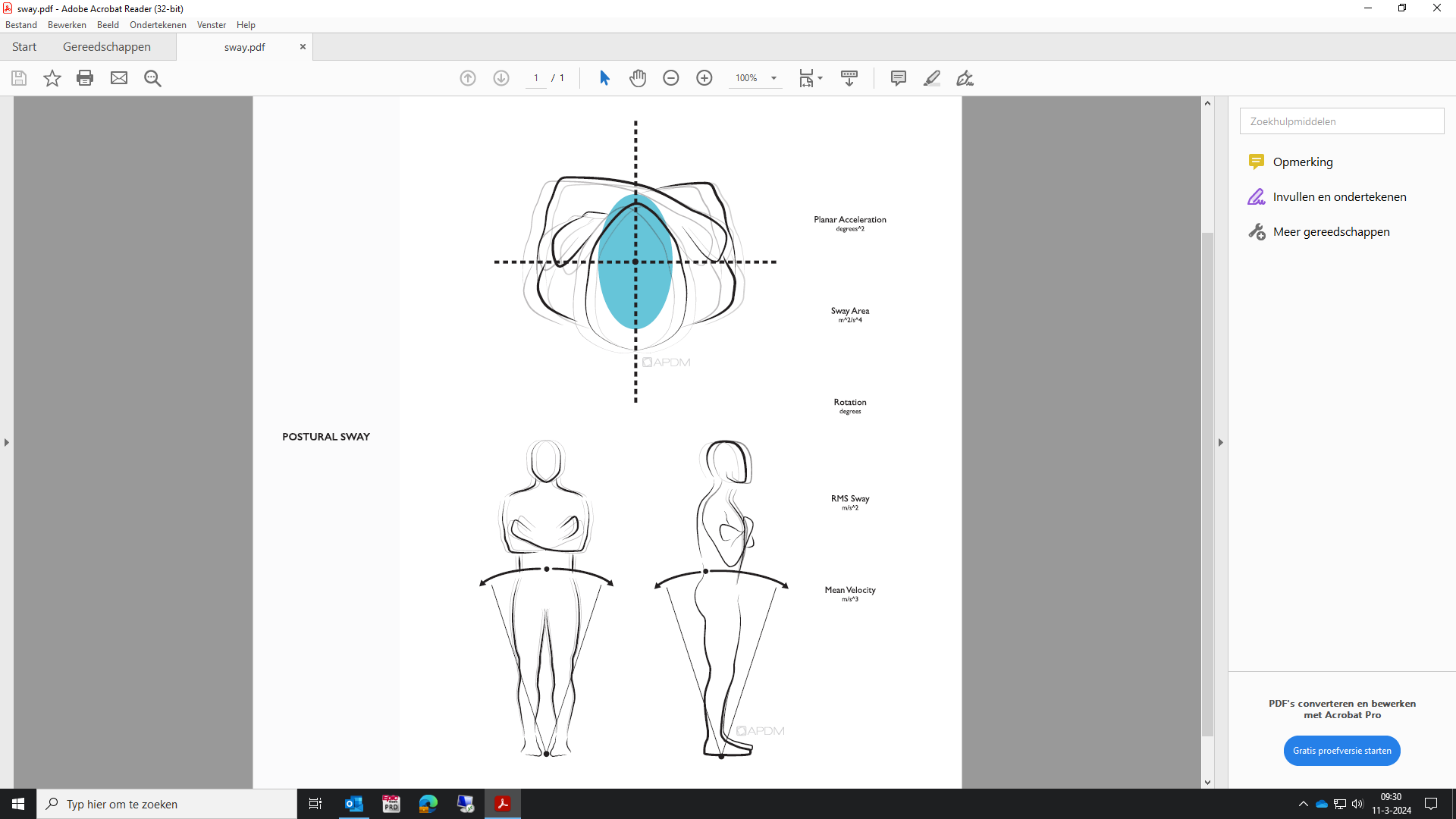


**Visual representation of Toe Off Angle**


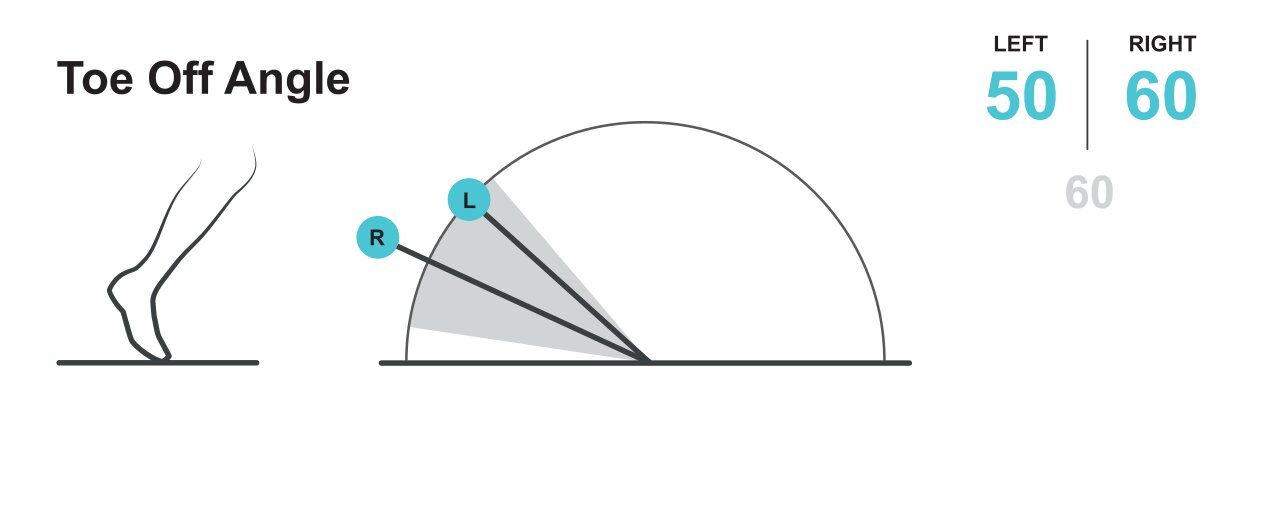


*Illustrations reprinted with permission from APDM an ERT Company 2019, Mobility Lab White Paper, retrieved from https://www.apdm.com/.*
